# Association Between Plasma and Urinary Reduced Thiols in Essential Hypertension: Evidence from a Paired Observational Study

**DOI:** 10.3390/jcm15031271

**Published:** 2026-02-05

**Authors:** Antón Cruces-Sande, Néstor Vázquez-Agra, Óscar Seoane-Casqueiro, Emma López-Prado, Estefanía Méndez-Álvarez, Ramón Soto-Otero, Antonio Pose-Reino, Álvaro Hermida-Ameijeiras

**Affiliations:** 1 Laboratory of Neurochemistry, Department of Biochemistry and Molecular Biology, Faculty of Medicine, University of Santiago de Compostela, 15782 Santiago de Compostela, A Coruña, Spain; estefania.mendez@usc.es (E.M.-Á.); ramon.soto@usc.es (R.S.-O.); 2 Health Research Institute of Santiago de Compostela (IDIS), Travesía da Choupana s/n, 15706 Santiago de Compostela, A Coruña, Spain; oscar.seoane.casqueiro@sergas.es (Ó.S.-C.); antonio.pose.reino@sergas.es (A.P.-R.); alvaro.hermida@usc.es (Á.H.-A.); 3 School of Industrial Engineering, University of Vigo (UVigo), 36310 Vigo, Pontevedra, Spain

**Keywords:** essential hypertension, oxidative stress, thiols, urine biomarkers, plasma biomarkers, redox status, non-invasive assessment, cardiovascular risk

## Abstract

**Background/Objectives:** Oxidative stress and extracellular redox alterations are involved in the pathophysiology of essential hypertension, but their clinical assessment is limited by the invasiveness and preanalytical complexity of blood-based measurements. Urine represents an attractive non-invasive biological matrix; however, the relationship between urinary and plasma DTNB-reactive reduced thiols in hypertensive patients remains insufficiently characterized. This study aimed to evaluate the association between plasma and urinary reduced thiols in essential hypertension. **Methods:** In this paired observational study, plasma and urine samples were obtained from 40 patients with treated essential hypertension. Reduced thiols were quantified using a DTNB-based colorimetric assay under identical analytical conditions. Plasma thiols were normalized to total plasma protein concentration, and urinary thiols were normalized to creatinine. Associations between plasma and urinary thiols were assessed using non-parametric correlation analyses. **Results:** Protein-normalized plasma thiols and creatinine-normalized urinary thiols showed a significant positive correlation (Spearman’s ρ ≈ 0.7, *p* < 0.001). **Conclusions:** In patients with essential hypertension, creatinine-normalized urinary reduced thiols are strongly associated with protein-normalized plasma reduced thiols, as measured by the DTNB reaction method. These findings provide hypothesis-generating evidence that urinary thiols may reflect extracellular thiol-related redox alterations, warranting further validation in independent and more diverse cohorts.

## 1. Introduction

Oxidative stress is a well-established consequence and modulatory component of the pathophysiology of essential hypertension and other chronic cardiometabolic conditions, reflecting disease-related alterations in the systemic redox state [1]. In human studies, circulating redox biomarkers are commonly used to approximate this state, because blood is accessible, standardized sampling is feasible, and plasma can integrate redox-related signals arising from multiple organs and exposures [2,3]. Nevertheless, plasma readouts represent proxies of a complex chemistry, and different biomarkers capture different facets of oxidative and electrophilic processes rather than a single ‘global’ variable, particularly in extracellular compartments [4,5].

Importantly, extracellular redox biomarkers should not be interpreted as reflecting a single equilibrated redox potential, but rather as operational readouts of partially coupled redox subpools maintained away from thermodynamic equilibrium by kinetic constraints, compartmentalization, and continuous fluxes of oxidants and electrophiles. This conceptual framework has been extensively discussed for plasma thiols, which constitute a dynamic but non-equilibrated network rather than a homogeneous redox system [5,6,7,8].

Among extracellular redox-related biomarkers, thiols occupy a central position because they act as major nucleophilic buffers and redox-responsive targets. The plasma thiol pool comprises low-molecular-weight thiols (e.g., cysteine, cysteinylglycine, homocysteine, glutathione) and a much larger reservoir contributed by albumin Cys34, which collectively participate in thiol–disulfide exchange and in reactions that buffer oxidants and electrophiles [5]. Albumin Cys34 alone accounts for approximately 70–80% of total reduced thiols in human plasma, whereas low-molecular-weight thiols circulate at much lower concentrations and are largely protein-bound [6,9,10]. Consistent alterations in thiol/disulfide balance have been associated with vascular dysfunction and adverse cardiovascular phenotypes across multiple settings, supporting the clinical interest of thiol-related measures as integrative indicators of extracellular redox perturbation [4,11].

Essential hypertension provides a particularly relevant clinical context to explore thiol-related biomarkers. Hypertension is highly prevalent and a leading modifiable contributor to cardiovascular morbidity and mortality, motivating continuous efforts to improve risk stratification beyond blood pressure values alone [12]. Mechanistically, oxidative stress is tightly linked to pathways implicated in hypertension, including endothelial dysfunction, vascular inflammation, and remodeling; while evidence is robust in experimental models, human translation remains heterogeneous, increasing interest in accessible biomarkers that can capture redox-related alterations in real-world patients [13]. In clinical cohorts, thiol/disulfide homeostasis has been reported to shift toward oxidation in primary hypertension and to relate to blood pressure and target-organ involvement, further supporting thiols as clinically meaningful readouts in this setting [14,15].

Despite this relevance, plasma thiol quantification is constrained by practical and preanalytical challenges, including the need for venipuncture, rapid handling to minimize artefactual oxidation, and normalization to protein concentration. These limitations reduce scalability for longitudinal monitoring and population-level applications. Urine offers an attractive complementary matrix: it is easily collected, enables repeated sampling, and is routinely normalized to creatinine to account for dilution [2,16].

Urinary biomarkers have been extensively explored as indicators of oxidative status in humans [17], including lipid peroxidation products, oxidized nucleic acids, and related end-products [18,19]. As discussed by Ilyasova et al. [2], these markers have proven informative at the population level despite the inherent particularities of renal handling, largely because urine offers a matrix with low organic and metal content—minimizing artifactual oxidation—and provides integrated indices of oxidative balance over longer time windows compared with blood-based measurements. Within this context, urinary redox biomarkers can be viewed as meaningful integrative readouts of extracellular oxidative and electrophilic pressures over time, while reflecting the extracellular milieu as sampled through kidney physiology rather than a direct mirror of plasma concentrations.

Importantly, urinary thiols reflect predominantly the low-molecular-weight fraction, because albumin is not filtered under physiological conditions; thus, urine provides a window into the smaller, more rapidly responsive thiol pool within the extracellular milieu [5,20]. In contrast to plasma—where low-molecular-weight thiols are largely bound to albumin and participate indirectly in redox buffering—urinary thiols are expected to represent freely soluble thiol species after renal processing [21]. This biochemical selectivity distinguishes urinary thiols from plasma measurements dominated by albumin-Cys34.

However, whether urinary thiol levels track plasma thiol status in a way that is clinically informative remains insufficiently characterized. Establishing a quantitative association between protein-normalized plasma thiols and creatinine-normalized urinary thiols would represent a first step toward evaluating urinary thiols as a non-invasive approach for thiol-related redox biomonitoring in hypertension. Therefore, in this study we examined the association between plasma and urinary reduced thiols in a paired cohort of patients with treated essential hypertension.

## 2. Materials and Methods

### 2.1. Design, Setting, and Participants

This was an observational paired-design study conducted at the Hypertension (HTN) and Cardiovascular Risk (CVR) Unit of the University Hospital Complex of Santiago de Compostela (CHUS) during the first semester of 2024. Patients aged ≥18 years with essential hypertension were consecutively recruited from routine clinical practice.

All participants underwent blood and urine sampling under identical conditions, with simultaneous measurement of circulating thiol-related redox biomarkers. The primary objective was to evaluate the association between plasma and urinary thiol measurements. As each participant served as their own plasma–urine comparison, no additional exclusion criteria were applied.

### 2.2. General Sample Variables

We collected data on participants’ age, sex, alcohol consumption (categorized as non-drinker vs. low-risk drinker), history of tobacco use (categorized as no/yes), and physical activity, following the European Society of Hypertension (ESH) guidelines [12]. Body mass index (BMI) was calculated as weight divided by height squared (kg/m^2^). Blood pressure levels were measured according to the STRIDE BP standards endorsed by the ESH [22]. The presence of arterial hypertension, diabetes mellitus, and cardiovascular disease was considered based on the definitions provided in the main consensus documents of the ESH, European Society of Cardiology (ESC), and American Diabetes Association (ADA) [12,23]. The use of antihypertensive medications and treatment adherence were also evaluated. Systematic use of high-dose antioxidant supplementation was not reported in the study cohort.

### 2.3. Sample Collection

Blood and urine samples were obtained at 08:00 h after a 12 h overnight fast, ensuring a minimum interval of 12 h since the last medication intake. Blood was drawn by peripheral venipuncture from the antecubital fossa using the vacuum method, applying less than one minute of tourniquet pressure, into EDTA-containing tubes (BD Vacutainer^®^, Becton, Dickinson and Company, Franklin Lakes, NJ, USA) [24]. Samples were centrifuged immediately at 1000× *g* for 10 min at 4 °C, and the plasma fraction was collected, aliquoted, and immediately transferred to dry ice prior to storage.

Urine samples were collected on site at the clinical unit at the time of the study visit, coinciding with blood sampling and under fasting conditions. First-morning voids were not required; instead, urine was obtained during the visit to deliberately minimize pre-analytical oxidation associated with prolonged overnight urine residence in the bladder and with inter-individual variability in specimen handling prior to processing. Although diurnal variability cannot be fully excluded, this approach was considered preferable for preserving thiol redox integrity, and creatinine normalization was applied to mitigate dilution-related variability. Urine was collected using a vacuum urine biochemistry tube (BD Vacutainer^®^, Becton, Dickinson and Company, Franklin Lakes, NJ, USA) and kept at 4 °C until centrifugation, which was performed within 15 min of collection at 1000× *g* for 10 min at 4 °C. The supernatant was aliquoted and immediately transferred to dry ice prior to storage.

Plasma and urine aliquots were stored at −80 °C for no longer than one month to preserve sample stability for analysis.

### 2.4. Assessment of Reduced Thiol Groups (Thiols) in Plasma (Thiol_p_) and Urine (Thiol_u_)

Ellman’s method employs 5,5′-dithiobis-(2-nitrobenzoic acid) (DTNB; catalog number D8130, ≥99%, Sigma-Aldrich^®^, St. Louis, MO, USA) to react with free sulfhydryl (–SH) groups, generating 5-thio-2-nitrobenzoate (TNB^−^), which can be quantified colorimetrically [25].

We prepared a 0.1 M sodium phosphate-buffered solution containing 1 mM EDTA, adjusted to pH 8.0. A standard calibration curve was generated using 1.5 mM L-cysteine hydrochloride (catalog number C7880, ≥98%, Sigma-Aldrich^®^) diluted in the buffer. The coefficient of determination (R^2^) for the calibration exceeded 0.98. Subsequently, we mixed 250 µL of buffer, 5 µL of DTNB reagent, and 25 µL of either standard or sample (in triplicate) in a microplate and measured absorbance at 412 nm after a 20 min incubation period using an Asys UVM-340 microplate reader (Biochrom^®^, Cambridge, UK).

This assay provides an operational measure of DTNB-reactive reduced thiols under standardized analytical conditions. Total plasma protein concentration was determined by the hospital central laboratory using a standardized automated biuret-based assay in routine clinical practice.

Thiol_p_ concentrations (µmol/L) were adjusted by total plasma protein levels (g/dL) and expressed as µmol/g protein (µmol/g Pp), in accordance with the literature [5]. Thiol_u_ concentrations (µmol/L) were corrected for urinary dilution using urinary creatinine concentrations (mg/dL) and expressed as µmol/mg creatinine, as commonly applied in clinical biomarker studies [16].

### 2.5. Ethics in Research

This study was conducted in accordance with the ethical principles of the Declaration of Helsinki and the Good Clinical Practice (GCP) standards of the Galician Health Service (Sergas). All patients who agreed to participate provided written informed consent. The Research Ethics Committee of Santiago–Lugo approved the study protocol (code 007/2023).

### 2.6. Statistical Analysis

The statistical analysis was performed using the SPSS 22.0 statistical program (SPSS Inc., Chicago, IL, USA), following a frequentist approach based on non-parametric statistics. The sample size was calculated using EPIDAT software, version 4.2 (Dirección Xeral de Saúde Pública, Xunta de Galicia, Santiago de Compostela, Spain), based on the objective of detecting a linear correlation coefficient of 0.5 between plasma and urinary thiols, with a 95% confidence level and a statistical power of at least 80% [26].

Extreme observations were winsorized to 1.5 times the interquartile range (IQR) above the third quartile and below the first quartile. The absence of missing values in the variables of interest was ensured.

A descriptive analysis was performed, and results were expressed as number (percentage) and median (interquartile range) for qualitative and quantitative variables, respectively. The association between plasma and urinary thiols was evaluated using Spearman’s rank correlation analysis. A logarithmic transformation of the variables was applied for graphical presentation of results.

Receiver operating characteristic (ROC) curve analyses were performed to explore the ability of urinary thiols to discriminate between low and high plasma thiol levels, using plasma thiol percentiles (P25, P50, and P75) as reference thresholds. Results were reported as area under the curve (AUC), sensitivity, and 1−specificity. A *p*-value < 0.05 was considered statistically significant.

In addition, a Bayesian bootstrap approach was applied to estimate the posterior distribution of Spearman’s rank correlation coefficient (ρ_s_) between plasma and urinary thiols [27]. A total of 5000 bootstrap replicates were generated using Dirichlet-distributed weights applied to the original observations. For each replicate, ρ_s_ was recalculated using weighted rank-based covariance. From the resulting posterior distribution, a 95% credible interval was derived, and a Bayes factor was calculated to compare the hypotheses H_1_: ρ_s_ ≥ 0.5 versus H_0_: ρ_s_ < 0.5 [28,29].

## 3. Results

### 3.1. General Features

**Figure 1:**
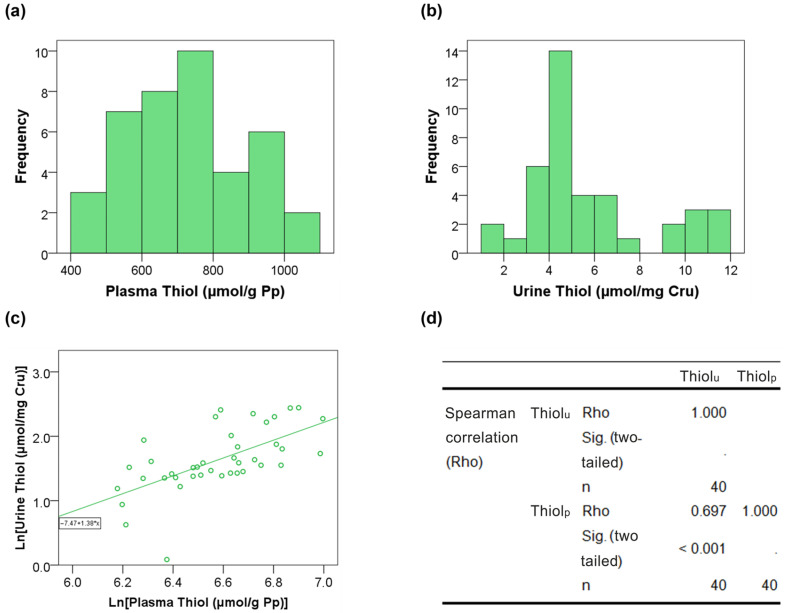
Statistical analysis of Thiol_p_ and Thiol_u_. (**a**,**b**) Histograms showing the distribution of the variables, both exhibiting non-normal distributions. Median and interquartile range (IQR) values are provided in Table 1. (**c**) Scatter plot of Thiol_p_ and Thiol_u_ after natural logarithmic transformation of both variables, shown for visualization of the monotonic association. The regression equation corresponds to the log–log representation and is not intended to imply linearity on the original scale. (**d**) Rank correlation analysis showing Spearman’s rho = 0.697, *p* < 0.001.

A total of 40 hypertensive patients (median age of 61 years) with full adherence to treatment, both in lifestyle habits and therapeutic regimen, were included. Of these, 22 (55%) were women. A total of 3 (7.5%) and 5 (12.5%) patients suffered from diabetes mellitus (DM) and cardiovascular disease (CVD), respectively. The general characteristics of the sample are detailed in Table 1. The distribution of the variables of interest is presented in Figure 1a,b. The median levels of plasma and urinary reduced thiols were 728.9 µmol/g Pp and 4.7 µmol/mg Cr_u_, respectively.

### 3.2. Analysis of the Thiol_p-u_ Relationship

**Figure 2:**
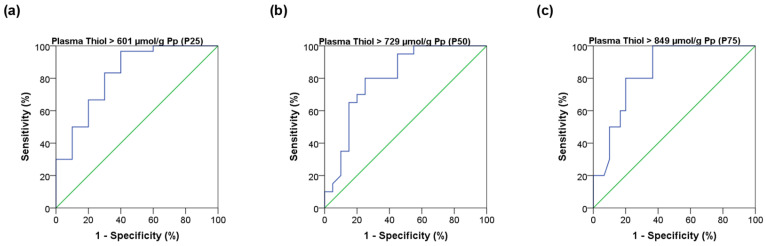
ROC curve models of urinary thiol levels (Thiol_u_, µmol/mg Cr_u_) for plasma thiol concentrations (Thiol_p_, µmol/g Pp) using specific cut-off points. (**a**) Thiol_p_ > 601 µmol/g Pp (P25): AUC = 0.823, SE = 0.082, *p* = 0.002, 95% CI: 0.662–0.984, Sensitivity = 0.83, 1–Specificity = 0.30; (**b**) Thiol_p_ > 729 µmol/g Pp (P50): AUC = 0.804, SE = 0.072, *p* = 0.001, 95% CI: 0.663–0.944, Sensitivity = 0.80, 1–Specificity = 0.30; (**c**) Thiol_p_ > 849 µmol/g Pp (P75): AUC = 0.842, SE = 0.062, *p* = 0.001, 95% CI: 0.720–0.964, Sensitivity = 0.80, 1–Specificity = 0.30. ROC—Receiver operating characteristic; Thiol_u_—Urinary thiols; Thiol_p_—Plasma thiols; Cr_u_—Urinary creatinine; Pp—Plasma protein. The blue line represents the ROC curve of the model, and the diagonal green line represents the line of no discrimination (AUC = 0.5).

Spearman’s rank correlation analysis showed a strong monotonic association (≈0.700) between Thiol_p_ and Thiol_u_ levels with the results provided in Figure 1c,d. Regarding the ROC curve analysis, Thiol_u_ levels showed an AUC close to 0.8, with 80% sensitivity for 25% false positives for Thiol_p_ levels higher than the median. The results for cut-off P25, P50, and P75 are shown in Figure 2.

### 3.3. Bayesian Inference

**Figure 3:**
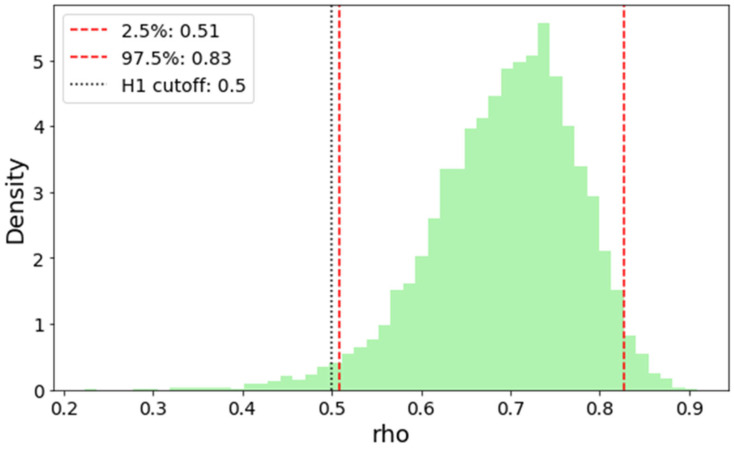
Posterior distribution of Spearman’s correlation coefficient (ρ_s_) estimated via Bayesian bootstrap. The histogram illustrates the empirical posterior density obtained from 5000 bootstrap replications, using Dirichlet-distributed weights applied to the original rank-transformed data. Vertical red dashed lines represent the 2.5th and 97.5th percentiles of the posterior distribution, forming a 95% credible interval for ρ_s_ (from 0.51 to 0.83). The dotted black vertical line at 0.5 marks the threshold used for hypothesis testing. The analysis yields an observed Spearman correlation of 0.697 and a Bayes Factor (BF_10_) of 44.45, indicating strong evidence that the true correlation exceeds 0.5. This visual and statistical summary supports a robust monotonic association between the paired variables. The Bayesian bootstrap approach used here avoids parametric assumptions and provides a full posterior distribution of the correlation coefficient, offering greater interpretability and reliability than conventional point estimates or *p*-values alone.

Bayesian bootstrap analysis yielded a posterior distribution concentrated around this value ρ_s_ = 0.697, as illustrated in Figure 3. The 95% credible interval for ρ_s_ ranged from 0.51 to 0.83, indicating substantial evidence for a moderate to strong monotonic association. The estimated Bayes Factor comparing the hypotheses H1: ρ_s_ ≥ 0.5 vs. H0: ρ_s_ < 0.5 was 44.45, indicating strong evidence that the true correlation exceeds 0.5. The posterior distribution was unimodal and approximately symmetric, further supporting the robustness of the observed correlation

## 4. Discussion

Urinary reduced thiols normalized by creatinine (Thiol_u_) showed a strong monotonic correlation with plasma reduced thiols normalized by total plasma protein (Thiol_p_; ρ ≈ 0.7, Bayes Factor > 40). This finding supports a close association between two analytically distinct but physiologically connected extracellular thiol compartments. Plasma-reduced thiols measured using DTNB predominantly reflect the redox state of albumin Cys34, the slow, high-capacity sink of the extracellular thiol–disulfide network [5,10,30]. In contrast, urinary thiols represent exclusively low-molecular-weight (LMW) thiols filtered and secreted into urine, comprising the rapidly responsive and electrophile-sensitive arm of extracellular thiol chemistry [20]. The observed association indicates that redox perturbations affecting these subpools tend to occur in parallel, allowing urinary LMW thiols to track changes in the broader extracellular redox environment.

Mechanistically, this relationship is coherent with the established organization of the extracellular thiol network. LMW thiol–disulfide couples and albumin-Cys34 remain kinetically linked via thiol–disulfide exchange reactions, which redistribute disulfide equivalents entering the plasma compartment. Oxidized disulfides—exported from tissues or generated extracellularly—together with electrophilic compounds, including downstream products of oxidative stress and exogenous xenobiotics—such as cigarette smoke [31]—initially engage the LMW thiol pool. LMW thiolates may either transfer oxidative equivalents onto albumin through thiol–disulfide exchange or be irreversibly consumed via covalent adduct formation with electrophiles [32]. The resulting pattern—oxidation and depletion of LMW thiols alongside accumulation of albumin mixed disulfides (Alb–SSX)—represents complementary biochemical consequences of shared upstream oxidative or electrophilic pressures. Our data suggest that these processes are sufficiently coordinated for urinary LMW thiols to track albumin-derived plasma thiol redox status.

The association between Thiol_u_ and Thiol_p_ remained consistent across multiple plasma thiol cut-offs, supporting its robustness for redox-based stratification within this clinical population. To our knowledge, this study provides the first quantitative in vivo evidence of a relationship between plasma and urinary reduced thiols in a human disease context. These findings highlight the practical utility of urine as a redox-informative biological matrix: its aproteic nature ensures that urinary thiols selectively reflect the LMW nucleophilic compartment, while creatinine normalization provides an established correction for urinary dilution.

From a translational perspective, plasma thiols offer mechanistically rich information but are constrained by venipuncture, stringent preanalytical handling, and protein normalization, limiting scalability in longitudinal or population-based studies [33]. In contrast, urine is non-invasive, inexpensive, easy to collect repeatedly, and routinely used in clinical practice [34]. The biochemical coherence demonstrated here suggests that urinary thiols may represent a feasible non-invasive marker of extracellular thiol-related redox alterations.

The Bayesian bootstrap analysis further strengthened these conclusions by providing an assumption-free inferential framework well suited to biomedical data [27,28]. Unlike parametric approaches, this method accommodates non-normal distributions and yields direct probabilistic evidence via Bayes Factors. The narrow credible interval and strong evidence favoring a correlation exceeding 0.5 underscore the robustness of the observed association and highlight the suitability of Bayesian approaches for translational redox research.

Despite these findings, the precise biochemical composition of urinary thiols warrants further investigation. Future studies should characterize the specific thiol species present (e.g., cysteine, cysteinylglycine, homocysteine, glutathione derivatives), their oxidation states, and their longitudinal behavior under defined oxidative or electrophilic challenges. Such work will help clarify the relative contributions of electrophile-driven thiol consumption versus disulfide-mediated redox redistribution, refining mechanistic interpretation of plasma–urine thiol coupling. Ultimately, linking these pathways to clinical outcomes may help establish urinary thiols as a scalable indicator of extracellular redox dynamics.

### Limitations and Strengths

This cross-sectional observational study has limitations inherent to its design and consecutive sampling strategy, including a heterogeneous patient population. Although the paired design—assessing Thiol_p_ and Thiol_u_ within the same individuals—minimizes inter-individual variability, the exclusive inclusion of patients with treated essential hypertension limits generalizability. Accordingly, the present findings should be interpreted as context-specific and hypothesis-generating, providing a conceptual framework that may motivate exploration of plasma–urine thiol coupling across other clinical conditions. Unmeasured confounders and random biological variability cannot be excluded.

As with any urinary biomarker, the relationship between plasma and urinary thiols is shaped by multiple layers of biological complexity that would be expected to weaken direct concordance between compartments. These include the intrinsically different biochemical nature of albumin-bound and low-molecular-weight thiol pools—characterized by distinct capacities, reactivities, and kinetics—as well as renal filtration, tubular handling, and potential intrarenal redox processes acting on the urinary compartment. From this perspective, the observation of a strong plasma–urine association is not trivial but rather noteworthy, as it emerges despite these intervening sources of variability.

In this cohort, a minority of participants met the definition of CKD (eGFR < 60 mL/min), but no patients with advanced renal impairment were included, and renal disease was not the dominant clinical phenotype. The dispersion observed around the fitted relationship is therefore expected and reflects biological variability inherent to the plasma–urine interface. Taken together, these considerations support the interpretation that urinary thiols capture, at least in part, a shared extracellular thiol-related signal, while also underscoring the need for caution when extrapolating these findings to populations with more severe renal dysfunction. The Thiol_p_ cut-offs applied (P25, P50, P75) were derived from the internal distribution of this dataset, reflecting the current absence of standardized reference ranges for extracellular thiol redox markers. Accordingly, these findings are not intended to support individual diagnostic use, but rather to inform redox stratification and exploratory screening within defined clinical contexts.

Furthermore, because Thiol_p_ and Thiol_u_ originate from distinct biochemical reservoirs, mechanistic interpretation remains provisional until urinary thiol species are chemically characterized. The observation of a robust plasma–urine association despite the multiple processes intervening between these compartments—including filtration, tubular processing, and potential intrarenal redox reactions—suggests coordinated regulation of extracellular thiol pools. Future studies combining thiol speciation analyses and controlled perturbations will be required to disentangle the relative contributions of these processes to the final urinary thiol signal.

Other strengths of the study include the paired design, rigorous preanalytical handling of plasma and urine samples, normalization to plasma protein and urinary creatinine, and the use of Bayesian bootstrap inference, which provides distribution-free robustness and enhanced interpretability for translational biomarker research.

## 5. Conclusions

This study demonstrates that creatinine-normalized urinary reduced thiols strongly correlate with protein-normalized plasma reduced thiols in patients with essential hypertension, as measured by DTNB reaction method. This association is robust across the observed range of values and persists despite the biochemical and physiological differences between plasma and urine compartments. The observed relationship is mechanistically plausible given the chemical interconnection between albumin-Cys34 and low-molecular-weight thiol pools via thiol–disulfide exchange and electrophile-responsive pathways.

By selectively reflecting the low-molecular-weight thiol fraction, urinary thiols provide complementary information to plasma measurements dominated by albumin-Cys34, while remaining aligned with the broader extracellular thiol redox environment. These findings position urine as a promising non-invasive matrix for assessing extracellular thiol redox status in a clinically relevant population.

Further validation in larger and more diverse cohorts, together with biochemical characterization of urinary thiol species and their relationship with relevant clinical outcomes, will be essential to define the clinical and translational utility of this approach and to clarify its applicability across different redox and disease contexts.

## 6. Patents

The authors report that part of the methodology used in this work is included in a European patent application filed by the Servizo Galego de Saúde, Universidade de Santiago de Compostela and IDIS. The patent application “IN VITRO METHOD FOR ASSESSING OXIDATIVE STRESS” was filed at the European Patent Office on 22 April 2025 under application number EP25382404.9 (submission number 300560808). The inventors listed are Antón Cruces-Sande, Néstor Vázquez-Agra, Álvaro Hermida-Ameijeiras, Estefanía Méndez-Álvarez and Ramón Soto-Otero.

## Figures and Tables

**Table 1:** General Features of the sample.

Variables	Total
	n= 40 ^a^
Age (years) †	61 (21)
Sex (women) ‡	22 (55)
Alcohol intake ^b^ ‡	6 (15)
Smokers ^c^ ‡	6 (15)
Physical activity ^d^ ‡	16 (40)
Diabetes ^e^ ‡	3 (8)
CKD ^f^ ‡	4 (10)
CVD ^g^ ‡	5 (13)
BMI (Kg/m^2^) †	29 (6)
SBP (mmHg) †	128 (21)
DBP (mmHg) †	76 (13)
HR (bpm) †	69 (12)
Antihypertensive drugs ‡	30 (75)
RAAS blockers ‡	25 (63)
Plasma creatinine (mg/dL) †	0.76 (0.3)
P_p_ (g/dL) †	7.2 (0.3)
Thiol_p_ (µmol/g P_p_) †	728.9 (247.8)
Cr_u_ (mg/dL) †	141.4 (64.0)
Thiol_u_ (µmol/mg Cr_u_) †	4.7 (2.8)

^a^ General description of the sample. ^b^ Low-risk alcohol consumption defined as less than 10 g/day for women and 20 g/day for men. ^c^ Current smokers or those who smoked within the past 6 months. ^d^ Moderate physical activity equivalent to 150 min of moderate-intensity walking per week. ^e^ Diabetes defined according to ADA criteria. ^f^ Defined as an eGFR below 60 mL/min. ^g^ CVD defined according to ESC criteria. Results expressed as † refer to the median and interquartile range, and results expressed as ‡ refer to the number and percentage. CKD—Chronic kidney disease; CVD—Cardiovascular disease; BMI—Body mass index; RAAS—Renin–angiotensin–aldosterone system; P_p_— Plasma protein; Thiol_p_—Plasma Thiols; Cr_u_—Urinary creatinine; Thiol_u_—Urinary Thiols; Kg—Kilogram; m—Meter; mmHg—Millimeter of mercury; bpm—Beats per minute; mg—Milligram; dL—Deciliter; µmol—Micromole; eGFR—Estimated glomerular filtration rate.

## Data Availability

The data supporting the findings of this study are available from the corresponding author upon reasonable request. Public deposition is not possible due to patient confidentiality constraints.

## Abbreviations

The following abbreviations are used in this manuscript:

ADA	American Diabetes Association
AUC	Area Under the Curve
BF	Bayes Factor
BF_10_	Bayes Factor (alternative vs. null)
BMI	Body Mass Index
CHUS	University Hospital Complex of Santiago de Compostela
CKD	Chronic Kidney Disease
Cr_p_	Plasma creatinine
Cr_u_	Urinary creatinine
CVD	Cardiovascular Disease
CVR	Cardiovascular Risk
DBP	Diastolic Blood Pressure
DM	Diabetes Mellitus
DTNB	5,5′-Dithiobis-(2-nitrobenzoic acid)
EDTA	Ethylenediaminetetraacetic acid
eGFR	Estimated Glomerular Filtration Rate
EH	Essential Hypertension
ESC	European Society of Cardiology
ESH	European Society of Hypertension
GCP	Good Clinical Practice
HR	Heart Rate
HTN	Hypertension
IQR	Interquartile Range
LMW	Low-Molecular-Weight
Pp	Plasma protein
RAAS	Renin–Angiotensin–Aldosterone System
ROC	Receiver Operating Characteristic
SBP	Systolic Blood Pressure
SE	Standard Error
STRIDE BP	STRIDE BP initiative for accurate blood pressure measurement
Thiol_p_	Protein-normalized plasma thiols
Thiol_u_	Creatinine-normalized urinary thiols
TNB^−^	5-Thio-2-nitrobenzoate
ρ	Spearman’s rho
ρ_s_	Spearman’s rank correlation coefficient
